# Nux Vomica

**Published:** 1889-05

**Authors:** F. L. Griffith

**Affiliations:** Edina, Mo.


### NUX-VOMICA.

EDITORS HOMŒOPATHIC PHYSICIAN:—I am induced by the idea that it will be of some benefit to Hahnemannians to say a few words in reference to Nux-vomica. We are told by the masters that it is a good idea to begin the treatment of a badly-drugged patient with Nux-v., that it will act powerfully as an antidote to allopathic or patent medicines, and leave you a clearer picture of *disease* without so much of the *drug* picture, and that it also often goes further even to the cure or partial cure of the case.

As I am the only homœopath (that I know of) in the north-east corner of Missouri, I have considerable experience with chronic patients who have withstood an enormous amount of drugging. I have followed the plan in numerous cases, and with astonishing success. When I get a bad old case I give Nux-v.$^{30}$ for four days, four doses a day. At the end of four days, I see the patient again and write up a second and altogether new history of the case, for which I prescribe according to the law.

We have repeated evidence that allopathy often defeats nature in her strenuous effort to throw off disease. I will give one case to illustrate.

Mr. ——, aged fifty-five, had been afflicted for six months with sciatica, and during the time took a great deal of strong medicine internally and various kinds of liniments applied externally. The disease was of a most tormenting nature. When I was called I gave Nux-v.$^{30}$, as above described, on account of the drugging he had had. I was greatly surprised in four days to find my patient almost well. The trouble got well "from above downward" (one of our famous laws of cure) but seemed to localize in the heel, for which I gave one dose of Sepia$^{cm}$.

My patient has been perfectly well for six months, and I got a great deal of credit for antidoting the medicine in the man and allowing him to *get* well—I say, *get* well, because I saw nothing in Nux-v. similar to his case. Nature was laboring for the cure, but allopathy and the disease combined were too much for her.

F. L. Griffith, M. D.

Edina, Mo.

